# Benzodiazepines II: Waking Up on Sedatives: Providing Optimal Care When Inheriting Benzodiazepine Prescriptions in Transfer Patients

**DOI:** 10.3390/jcm7020020

**Published:** 2018-01-30

**Authors:** Jeffrey Guina, Brian Merrill

**Affiliations:** 1 Department of Psychiatry, University of Michigan Medical School, Ann Arbor, MI 48109, USA; 2 Department of Psychiatry, Wright State University Boonshoft School of Medicine, Dayton, OH 45417, USA; brian.merrill@wright.edu

**Keywords:** benzodiazepine, sedative, hypnotic, anxiolytic, psychopharmacology, alprazolam, clonazepam, evidence-based, taper, monitor

## Abstract

This review discusses risks, benefits, and alternatives in patients already taking benzodiazepines when care transfers to a new clinician. Prescribers have the decision—sometimes mutually agreed-upon and sometimes unilateral—to continue, discontinue, or change treatment. This decision should be made based on evidence-based indications (conditions and timeframes), comorbidities, potential drug-drug interactions, and evidence of adverse effects, misuse, abuse, dependence, or diversion. We discuss management tools involved in continuation (e.g., monitoring symptoms, laboratory testing, prescribing contracts, state prescription databases, stages of change) and discontinuation (e.g., tapering, psychotherapeutic interventions, education, handouts, reassurance, medications to assist with discontinuation, and alternative treatments).

## 1. Introduction

Benzodiazepines (BZDs) are some of the most commonly prescribed medications in the world [1,2]. The risks and benefits of these medications have been widely debated [3,4]. Many patients take BZDs long-term without ever receiving evidence-based first-line treatments such as psychotherapy or serotonergic agents. Many clinicians are very cautious in prescribing BZDs, and some even have clinic policies against their prescription, but it is not uncommon for these same clinicians to have patients transferred to them from other clinicians who are less reluctant to prescribe BZDs. Many clinicians are comfortable continuing BZD prescriptions in some patients for months or even years and, when these patients transfer to a new clinician who is loath to prescribe BZDs, it can put the gaining clinician in a quandary. Is it the new prescriber’s responsibility to continue the prescription? To provide a taper? Or, is the clinician legally and ethically able to refuse to continue the prescription they did not start and do not think is indicated, even if it risks withdrawal? This review discusses risks, benefits, and alternatives in patients already taking BZDs when transferring care to a new clinician.

A few years ago and after treating several patients with posttraumatic stress disorder (PTSD) who were “inherited” from other providers with BZD prescriptions, the authors of this study began questioning if any good was being done by continuing the prescriptions. These patients had long histories full of distress, dysfunction, and hopelessness. Moreover, they did not seem to improve like other PTSD patients. The authors began hypothesizing that the medications may be inhibiting improvement. Which came first: the severity and chronicity, or the BZD? Many of these patients had been taking BZDs for decades, many of them had never been treated with another medication class or psychotherapy, and almost all of them firmly believed that their PTSD could never improve. When provided education about prognosis and evidence-based treatments, most were reluctant to change and some even reacted with hostility when it was suggested that other treatments could improve their lives. Those that accepted evidence-based treatments almost universally improved.

If avoidance is a poor prognostic factor for anxiety and BZDs are inherently avoidance-producing (i.e., inhibit cognitive processing and promote numbing), could BZDs prolong or worsen anxiety? Due to frequent resistance to alternatives and many claims that BZDs were helping (despite the persistence of severe symptoms), the authors were reluctant to change treatments against patients’ desires. When there was evidence of harm (e.g., excessive sedation, falls, delirium) or misuse (i.e., use other than as prescribed—e.g., presence of illicit substances in urine-drug-screens, prescriptions from multiple providers, early refills), treatment changes were often made unilaterally. For others, the authors were reluctant to make changes based on instinct and anecdote without a basis in evidence. When initially looking for evidence about BZDs for conditions like PTSD, it was common to find articles lamenting the lack of studies on the subject, or recommendations for or against BZDs without referencing studies. However, there are several studies in existence, and we review them here.

## 2. Deciding the Recommendation

A particularly challenging clinical scenario occurs when one encounters a patient that has been maintained on BZDs for an extended period of time (months to years). In these situations, the clinician must first assess the need for ongoing BZD maintenance. If it is determined that the patient is at risk of adverse consequences of BZDs or that the indication for benzodiazepines no longer exists, then weaning is prudent.

While the decision to forgo initiating a BZD in favor of evidence-based first- and second-line treatments is easy, it can be very difficult to decide how to manage a patient transferred from another prescriber who was prescribing BZD. BZD dependence is preventable with short-term use (i.e., less than 2–4 weeks) [1], but even more preventable by using other of the various medications for anxiety. Once BZDs are used, patients are often reluctant to change medications or may not find replacement medications as subjectively pleasing. Prescribers have the decision—sometimes mutually agreed-upon and sometimes unilateral—to continue, discontinue, or change BZDs. This decision should be made based on evidence-based indications (conditions and timeframes), risk of adverse effects and misuse, comorbidities, and potential drug-drug interactions.

This decision should not be taken lightly. Continuing a harmful treatment violates the primum non nocere principle, but removing a treatment perceived as helpful could risk the therapeutic alliance. Some prescribers may oppose continuing BZDs even when the potential for harm is high even in the absence of adverse effects or misuse, while others argue for vigilant monitoring. Some prescribers may push for unilaterally discontinuing BZDs when contraindications are present but no definitive harm, though others may decide to continue BZDs when patients protest their discontinuation all the while providing psychoeducation and working through the stages of change to move a patient away from BZDs and towards evidence-based treatments. “Unwilling patients should not be forced to withdraw. It is unwise and unkind to compel withdrawal. Enforced withdrawal is usually unsuccessful and leads to unnecessary distress” [5]. Nevertheless, many clinicians do not want to assume the risks and responsibilities associated with continuing to prescribe BZDs.

There are great ethical concerns and legal liability/criminal responsibility [6] when BZDs are continued in the presence of clinical risks, but there are also great clinical and ethical concerns when unilaterally discontinuing medications against a patient’s wishes. These include withdrawal, but also simply losing a disgruntled patient, either preventing them from getting any care, leading them to seeking self-treatment from illegal sources, or leading them to seek care from a less scrupulous or less evidence-based prescriber. Seeking BZDs elsewhere and relapse rates after BZD discontinuation range from 8–57% [1,7,8,9]. “Quality patient focused care involves weighing up the balance between the modest benefits of benzodiazepines with the substantial risks and harms in some patients” [10]. Prescribers must weigh the risks of continuing treatment (e.g., adverse effects, misuse, and delaying definitive treatment and recovery) and the risks of discontinuing treatment (e.g., withdrawal, fleeing treatment, seeking inappropriate alternative sources).

With patients taking BZDs, the initial goals of treatment should be to determine diagnosis and to establish a therapeutic alliance. “The art of treatment is knowing when the therapeutic alliance is sufficiently established to institute drug withdrawal, and knowing when outpatient treatment is not progressing adequately” [11].

### 2.1. Assessing Proper Indication

In patients taking BZDs, providers need to make an accurate diagnosis. This includes differentiating primary disorders from BZD-induced disorders, and eliciting a thorough course of illness via interview and collateral sources (certain symptoms may have worsened or only developed after initiation of BZDs). Once an accurate diagnosis is made, the patient’s treatment history can be assessed to determine if they received adequate courses of first- and second-line evidence-based treatments for that diagnosis. If the variety of evidence-based treatments were not exhausted before BZDs were initiated (e.g., a PTSD patient tried prolonged exposure (PE) but not cognitive processing therapy (CPT) or eye movement desensitization and reprocessing therapy (EMDR), and a selective serotonin reuptake inhibitor (SSRI) but not a serotonin norepinephrine reuptake inhibitor (SNRI)), adding or changing to unexplored evidence-based treatments should be encouraged.

### 2.2. Assessing Effectiveness

Frequently, both clinical and research assessments of improvement of mental disorders are based on self-reported levels of symptoms. However, most clinicians, researchers, and patients would agree that level of functioning is more important than level of symptoms. Anxiety and insomnia are very simple symptoms to treat: one only need provide enough of a central nervous system (CNS) depressant—whether diazepam, midazolam, fentanyl, alcohol, or heroin—until someone is calm or asleep. This is not functional recovery. Many patients report a sedative “helps” their anxiety, yet their lives are no better. The aims of clinicians should not be to treat anxiety but to treat functional impairment. If a rare BZD allows an individual to fly on a plane, or if someone was unable to sustain steady employment before BZDs and has steady employment since being prescribed BZDs, then BZDs are effectively treating impairments in functioning. However, if despite BZDs, they continue to have difficulty leaving their home, working, and interacting with their family, than BZDs may be effective for anxiety but they are not effective for their functioning. In the absence of obvious harms, the presence or absence of effectiveness should be the principal determinant of BZD continuation or discontinuation.

### 2.3. Assessing Harms

When patients present with problems, clinicians’ first instinct is to be active—to provide or increase treatment rather than remove or decrease treatment. However, one of the first questions a clinician should always ask themselves is, “what have other providers or I done to potentially cause this” before ruling in or ruling out iatrogenic effects and proceeding with assessment. After all, “first, do no harm.”

A thorough history, review of systems, review of collateral information, and laboratory testing (e.g., for the presence of other substances which indicates misuse or the absence of BZDs which indicates diversion) should be used to determine the presence of adverse effects (which patients often fail to connect to their medications on their own), contraindicated conditions and medications, and misuse. Signs that patients are not appropriately using prescribed BZDs include the fabrication of psychiatric or medical symptoms, signs of intoxication, using BZDs more than prescribed, early refills, doctor shopping (which is most easily verified by prescription monitoring systems), negative blood or urine tests for BZDs (indicative of diversion), and positive tests for other substances.

Contraindications, significant adverse effects, drug-drug interactions, and evidence of misuse (including diversion) should lead to unilateral discontinuation (by outpatient taper, inpatient admission, or immediate discontinuation, when appropriate). Minor adverse effects or physical dependence (i.e., tolerance and/or withdrawal) in the absence of misuse should lead the prescriber and patient to engage in discussions that weigh the risks and benefits of continuing BZDs, or changing to more effective and/or safer alternatives (see Table 1) [12].

## 3. Continuation

Though steeped in controversy and clearly not without potential hazards, BZDs remain viable treatment options for multiple anxiety disorders and insomnia. Below we will discuss relevant evidence for the use of benzodiazepines and provide practical guidance in deploying these agents in the treatment of anxiety and insomnia. Any prescription of BZDs—including initiation and continuation—should be preceded by thorough discussion of possible risks and benefits, including the potential for abuse (i.e., continued use despite harms) and dependence.

When patients refuse evidence-based treatment trials and the benefits are deemed to outweigh the risks, clinicians at times choose to continue prescribing BZDs outside of evidence-based and/or FDA-approved indications. Physical dependence on BZDs may be acceptable if psychosocial functioning is improved with BZDs due to the effective treatment of disabling anxiety [11]. However, continued monitoring, education, and the use of additional treatments should be utilized. Tannenbaum et al.’s EMPOWER (Eliminating Medications Through Patient Ownership of End Results) trials have demonstrated that when patients are educated about the potential harms of BZDs, they often want to taper from them [13,14]. The authors have frequently observed this phenomenon in their own clinical experience.

### 3.1. Monitoring

When a prescriber makes the decision to continue BZDs, there are a variety of steps they can and should consider. Close patient monitoring is recommended [15]. Clinical and risk management tools should be used on a regular basis, and not just the first time BZDs are prescribed.

Regular education is important to assure proper use of BZDs. For example, elderly patients should regularly be warned about the increased risk with BZDs for falls, hip fractures, cognitive impairment, and dementia that can persist beyond BZD discontinuation [15,16,17]. All patients taking BZDs should regularly be advised not to use alcohol on the same day (or even within days or weeks for elderly patients or those with hepatic impairments) [7,8,9,15,18]. Prescribers should warn patients about the risks of driving while taking BZDs. Prescribers should advise all patients of the risk of dependence, adverse effects, and drug-drug interactions (including regular reminders and regular assessments of medications prescribed by other sources).

The easiest and likely most common tool is simply monitoring symptoms and vital signs. Prescribers should regularly assess for stability of underlying symptoms, development of adverse effects, and functional impairment. Functional impairment is often under-evaluated, but is arguably the most important marker of treatment effectiveness. Clinicians often tend to focus on self-reported subjective distress rather than objective dysfunction. It is important to eliciting levels of, changes of, and examples about various domains of functioning: academic, occupational, social, interpersonal, recreational, effective communication, self-care, and safety. Optimization of functioning is more indicative of recovery and treatment effectiveness than reduction of symptoms. Additionally, remission and not simply symptom management (e.g., daily anxiety attacks aborted with BZDs with reoccurrence each day).

Some providers find contracts to be helpful tools with patients when using agents with high potential for abuse and addiction such as BZDs. These contracts can specify parameters for both parties and serve to codify expectations in the doctor-patient relationship as they relate to the prescribed medication and other elements of the treatment plan (e.g., attending psychotherapy aimed at addressing anxiety). Prescribers should advise all patients of the risk of dependence, adverse effects, indication(s) for continued use, that the lowest dose for the shortest period of time to achieve the desired outcome will be provided, that they will be monitored closely (e.g., weekly reviews, urine drug screens), that the treatment of anxiety and insomnia should rely largely on nonpharmacological interventions (e.g., psychotherapy, sleep hygiene, relaxation techniques), and that they must obtain all prescriptions from the same provider and pharmacy (and inform the provider of changes) [10]. These agreements should be clearly explained to the patient who signs the agreement, a copy is provided to the patient, and it is clearly documented in the record.

Laboratory tests (e.g., blood and/or urine) can help detect mixing of drugs and diversion. Potential pitfalls include false positives/negatives and knowing which specific drugs are tested for (e.g., it is common for “benzodiazepines” to only refer to diazepam, temazepam, and oxazepam; alprazolam and clonazepam are high-potency BZDs commonly preferred by people with substance use disorders (SUDs) but are often undetected in drug screens due to being excreted in small amounts in urine; long half-life BZDs like diazepam and chlordiazepoxide that are taken long-term or in high doses may be detected for weeks). Different laboratories have different cut-offs, detection times, and drugs included in screening panels [11]. For these reasons, prescribers should consult their laboratories directly to learn about variations and obtain lists of specific drugs tested and of potential false positives/negatives for each drug. Additionally, the use of quantitative—rather than qualitative—testing (universally or only on samples for which the qualitative drug screen is positive) can confirm that the initial screen was positive, and can differentiate specific drugs and metabolites. However, some laboratories may dispose of the sample before a quantitative test can be performed. Considering the risk and prevalence of mixing BZDs and alcohol, blood alcohol tests (which are subject to zero-order metabolism) and urine ethylglucuronide/ethylsulfate (which detects ethanol metabolites 1–3 days following use, similar to most urine drug testing). Toxicology tests can be useful to confirm substance use.

In addition to laboratory tests, another tools to detect misuse, diversion, and doctor shopping is prescription databases. Some states (e.g., Ohio) require all prescribers to check their state’s controlled substance database at the time of every controlled substance prescription. These systems can be useful for determining what controlled substances a person has access to, how much is dispensed, how many prescribers are dispensing, and frequency of refills. Potential pitfalls include patients who cross jurisdictions (e.g., multiple states, federal systems like the Department of Defense and/or Veterans Affairs) for which all of a particular patients’ prescriptions may not be recorded in a specific database (although many states share data).

Just as important as education about proper usage is education about gold standard treatments and the lack of evidence for long-term BZDs. Just as with SUD patients, prescribers should frequently assess BZD patients for readiness for change using the change continuum (e.g., pre-contemplation, contemplation, preparation, action, maintenance), and work towards moving the patient away from long-term BZDs and towards evidence-based treatments.

### 3.2. Changing Benzodiazepines

Expected onset and duration of action and metabolic properties for individual patients are important considerations when selecting amongst particular benzodiazepines. Considerations should be based on the indication, pharmacokinetics, and pharmacodynamics of each particular agent. The pharmacodynamic considerations for prescribing benzodiazepines are largely relevant in the context of abuse and addiction, as higher potency and shorter-acting agents have higher potentials for abuse.

Even within the individual disorders, the clinical phenomena of anxiety can be episodic or continuous (e.g., a patient with generalized anxiety disorder (GAD) can experience episodic intense worry with an increase in somatic symptoms and a patient with panic disorder (PD) can experience continuous worry about another panic attack). BZD regimens should be selected so that the pharmacokinetic properties of the selected agent correspond to the expected duration of symptoms. For example, continuous, gnawing anxiety that lasts all day may be best treated with longer-acting, whereas briefer and more pulsatile anxiety experiences might be best treated with short- or intermediate-acting agents. APA guidelines suggest that long-term treatment with BZDs is helpful to some with PD, though this is based on clinical experience, as opposed to randomized controlled trials or other rigorous study designs [19].

### 3.3. Adding Psychotherapy

Psychotherapy is the gold standard treatment for anxiety (including PTSD) and insomnia. Psychotherapy is a “mainstay in the treatment of all anxiety disorders; exposure to feared situations is necessary to move beyond phobic avoidance and functional impairment to full recovery, the ultimate goal of therapy” [20]. BZD monotherapy is not recommended in anxiety disorders nor PTSD, but may be useful when very anxious PTSD patients have poor responses with other medications [15]. However, in these cases, it is incumbent on providers to recommend psychotherapy as a potential alternative or—at least—additional treatment. This is important not just for those prone to medication side effects, but for all patients. Just as stress and trauma can negatively change the brain, neuroimaging studies demonstrate that psychotherapy can positively change the brain [21,22]. “The most effective and promising therapeutic approach to PTSD employs medication in combination with cognitive behavioral interventions” [23]. In fact, many would argue that medication alone—without psychotherapy—is not appropriate treatment for anxiety or insomnia.

Prescribers who do not provide psychotherapy themselves should refer patients for psychotherapy that is evidence-based for the indicated condition. Cognitive behavioral therapy (CBT), including exposure therapies such as systematic desensitization and flooding), psychodynamic, acceptance, and commitment therapy, mindfulness-based, relaxation, feedback, interpersonal, assertiveness training, and dialectical behavioral therapies are each efficacious for anxiety [24,25,26,27]. These and trauma-focused psychotherapies (e.g., CPT, PE, EMDR) are efficacious for trauma/stressor-related disorders such as PTSD [28,29]. For insomnia, CBT, CBT-I, stimulus control, relaxation, and sleep restriction are efficacious [30]. While not every type of evidence-based therapy are accessible in all areas, there is a high likelihood that at least one of these modalities will be available. For those with limited funds/insurance, free clinics, community mental health clinics, and universities/colleges/training programs (e.g., psychiatry, psychology, clinical social work, licensed counselor) may be sought out.

Unfortunately, BZDs can inhibit the therapeutic effects of psychotherapy by promoting avoidance, inhibiting cognitive processing, and inhibiting fear extinction upon exposure [31,32,33,34,35]. Nevertheless, most clinicians agree that it is better to provide psychotherapy to patients who refuse to stop BZDs—even if they will have more modest effects—than to deny treatment.

### 3.4. Adding Additional Pharmacotherapy

Psychotherapy is the preferred first-line treatment for anxiety disorders, but pharmacotherapy is also an important treatment option, especially when there is limited availability of psychotherapy or to comply with patient preference. Pharmacotherapy can not only improve symptoms, but in an empathic setting, can improve patient engagement in their treatment [36]. While BZDs are effective for some anxiety disorders, other medications such as serotonergic agents are more effective and safer, especially in the long-term. When patients refuse to stop BZDs and prescribers deem that it is safe to continue them, BZD monotherapy should be avoided. In addition to psychotherapy, evidence-based medication trials should be used to improve symptoms and help work towards transitioning away from long-term BZDs—due to lack of efficacy and significant risks. The downsides of prescribing BZDs with SSRIs is that there is a greater risk of drug–drug interactions with multiple medications (e.g., synergistic sedating effects) and it can make it difficult to discriminate side effects that may occur from each medicine [15]. The benefit of starting another medication in the presence of BZDs is that there is typically less resistance to, and less likelihood of attributing withdrawal/rebound symptoms to, the evidence-based medication.

Table 1 displays some evidence-based alternatives for BZDs for both short-term/fast-acting and long-term uses. Serotonergic agents (e.g., SSRIs, SNRIs, tricyclics, mirtazapine, monoamine oxidase inhibitors, trazodone, nefazodone, buspirone) have the strongest evidence of greater therapeutic effects and less adverse effects than BZDs, but adrenergic inhibitors (e.g., propranolol, prazosin, clonidine, guanfacine), antihistamines (e.g., hydroxyzine, diphenhydramine), anticonvulsants (e.g., gabapentin, pregabalin, lamotrigine, topiramate, valproate), antipsychotics (e.g., quetiapine, olanzapine, risperidone), memantine, and triiodothyronine all have stronger evidence for certain anxiety/insomnia disorders than BZDs [25,28,29,36,37,38,39,40,41,42,43,44,45]. Consideration for unique adverse effects and comorbidities should be given. For example: hydroxyzine may be particularly effective for people with anxiety, insomnia, nausea/vomiting, and/or allergies (all indications); amitriptyline may be particularly effective for people with anxiety, insomnia, depression, neuralgias, and/or migraines (all indications); clonidine may be particularly effective for people with anxiety, insomnia, inattention ± attention-deficit hyperactive disorder, tics, cancer-related pain, and/or hypertension (all indications); lamotrigine may not be first-line, but may be used earlier in the course of successive medication trials for people with anxiety, bipolar disorder (especially bipolar depression), and epilepsy (both indications), and/or obesity (lack of weight gain effects); and it may be prudent to avoid (or try later in the course of medication trials) olanzapine in those with obesity and/or diabetes (metabolic risks), but useful to try it earlier in those with anxiety, insomnia, depression, psychosis, and/or bipolar disorder. 

Psychological and behavioral therapies are standard for insomnia (e.g., stimulus control therapy, relaxation therapy, CBT/CBT-I, sleep restriction therapy, sleep restriction, paradoxical intention, biofeedback therapy) [30]. However, some pharmacological treatments are weakly evidence-based. Non-BZD gamma-Aminobutyric acid (GABA) receptor agonists (i.e., zolpidem, zaleplon, eszopiclone) have weak evidence for insomnia, but have similar risks as BZDs [1,46]. The non-sedative-hypnotics with the strongest—though still relatively weak—evidence for insomnia include: ramelteon, trazodone, amitriptyline, doxepin, mirtazapine, gabapentin, tiagabine, quetiapine, and olanzapine [30]. Sedating antidepressants are especially encouraged when used in conjunction with depression/anxiety [30].

Regardless of which medications are prescribed (BZDs and/or anything else), prescribers should recommend medications “preferably in combination with non-pharmacological treatments such as cognitive behavioral therapy” [38] because appropriate care “involves not only providing the best initial evidence-based pharmacotherapy, but also ideally delivering it collaboratively with a psychotherapist” [36]. Comprehensive treatment approaches involving both psychotherapy and pharmacotherapy are especially important for chronic treatment-refractory anxiety and insomnia patients.

## 4. Discontinuation

Mental health treatment should aim to reduce symptom severity, prevent and treat comorbid disorders, decrease functional impairment, modify pathogenic fear schemas, prevent relapse, build resilience, and improve the quality of life [45]. Often, prescribers focus on the first goal to the detriment of the others. When patients are self-reporting symptom reduction with BZDs, but have evidence of adverse effects, misuse, contraindicated comorbidities (e.g., depression, SUDs, neurocognitive disorders) and/or—most importantly—persistent functional impairment despite of or because of BZDs, many prescribers will choose to cease BZD prescriptions even against patients’ requests. Discontinuation of BZDs is appropriate for those experiencing breakthrough symptoms that were previously well-controlled, neurocognitive side effects, and abuse of other substances (e.g., alcohol, cocaine, other prescription medications) [11].

Decreasing and discontinuing BZDs can be terrifying for patients. Approximately one-third of patients have difficulties during managed BZD withdrawal [5], and even more likely have high anticipation anxiety even before starting the discontinuation process. It is difficult to study BZD discontinuation because of high rates of patients refusing to participate in and of dropping out of BZD withdrawal studies [1]. This is a testament to how powerful BZD dependence is. Dropping out of a BZD-tapered discontinuation study is associated with not being married, short-acting BZDs and extraversion. Successful discontinuation is associated with low baseline neuroticism, low behavioral inhibition due to anxiety, higher number of positive life events, and higher level of social support satisfaction. Self-efficacy in coping during the tapering period is associated with decreased relapse. “Psychological differences might explain why some patients do and others do not experience difficulties stopping BZDs” [47]. Even with education, it is often difficult to transition patients from BZDs—which they have potentially been using for years—to evidence-based treatments. Psychotherapeutic interventions and replacement pharmacotherapy to target the symptoms which these patients are most afraid of can be helpful in the transition.

While symptom relapse after BZD discontinuation is high only for a brief period of time during acute withdrawal, there is a high risk of relapse—perhaps as high as 50%—on BZDs often with patients seeking the medications from other prescribers and/or illegal sources [8]. Relapse (i.e., resumed use) after BZD discontinuation is associated with abrupt withdrawal (i.e., without taper), insomnia severity, psychological distress (e.g., anxiety, major life changes such as divorce, retirement, or death of a spouse), snoring partner, hospitalization, or surgery and chronic pain [8]. Elderly patients are significantly less likely to stop BZDs than younger patients. Many do not see an advantage to withdrawal and believe BZDs are effective. However, the cognitive advantages to withdrawal and lack of effectiveness of long-term BZDs may be persuasive [7]. To reduce relapse risk, there are several tools to assist in discontinuation: education, handouts, psychotherapeutic interventions, tapering, medications to assist with discontinuation, and alternative medications. The keystones of BZD discontinuation are gradual tapering, psychological support, and judicious individual management [1]. When BZD discontinuation is managed judiciously and individually, success rates can be 70–80% [1,7,48].

### 4.1. Education and Support

Education and handouts can improve successful discontinuation. Prescribers should continuously discuss the proper indications of BZDs, the alternative evidence-based indications, and the risks of continuing BZDs. Because it is difficult to determine whether or not distress and dysfunctional behaviors are the result of BZDs, and because providers, patients, and patient families may disagree on the cause of these symptoms, the patient may need to be observed medication-free for a time. Symptoms may then be reframed as the result of “addictive medications” or “dependence” rather than the underlying psychiatric disorder [11]. Prescribers should prepare patients for rebound/withdrawal symptoms, reassure that anxiety/insomnia will improve after days–weeks, and make a plan to work towards reducing any residual anxiety/insomnia with evidence-based treatments. Written withdrawal schedules and information sheets are more effective than verbal instructions. Calendars or charts allow patients to record their progress, increasing incentive to continue. Useful support of patients with withdrawal anxiety includes written information about what to expect with BZD withdrawal aimed at patients (including books and pamphlets, prescriber-screened website referrals, and material individualized by the prescriber for the patient), keeping a diary to pinpoint precipitating factors and other alternative explanations for panic attacks other than simply the absence of BZDs, and reassurance [1,5]. Protocols for BZD-related education based on randomized trials are available [13,14]. Several studies (including randomized controlled trials) have demonstrated that primary care consultations and receiving a letter signed by a primary care provider advising gradual reduction in BZD use can facilitate BZD discontinuation [49]. It may be helpful to explain BZD physical dependence and discontinuation as processes of “neuroadaptation” [11].

Since patients “remain vulnerable to stress for some months” after BZD discontinuation [1], individual support via the therapeutic relationship can greatly improve treatment adherence and successful transitions towards evidence-based treatment. BZD withdrawal is most likely to be successful with a combination of gradual dose reduction and psychological support. When managed well, success rates for stopping BZDs are approximately 90%, with the majority feeling better after withdrawal than when they were taking BZDs [5]. Psychological support should be available both during tapering and for some months after discontinuation. Some patients may do better with weekly contact—by phone and/or face-to-face—in the initial stages, usually to assess causes of anxiety and provide reassurance. Support includes provision of information about BZDs, general encouragement, and psychotherapeutic interventions. Useful support of patients with withdrawal insomnia includes reassurance, social support, referrals to support organizations and 12-step recovery programs, family psychoeducation, accurate evaluation of the social and interpersonal context, physical examination, sleep hygiene, relaxation techniques, and constant telephone availability of a clinician [1,5,18,48]. Support organizations and self-help groups such as 12-step recovery programs (e.g., Narcotics Anonymous, Alcoholics Anonymous) are important treatment adjuncts. Providers should warn patients that groups vary significantly in philosophy, with some groups opposing the use of all psychotropic medications [11]. Self-help groups run by ex-BZD users may be helpful, but psychotherapy is more effective, especially when individual rather than group psychotherapy [5]. Involving spouses or family can increase support. Many patients only require minimal support, though a few may need formal psychotherapy. During withdrawal, it is important that patients are provided individual counseling with specific advice, options for self-help support programs, and referrals when necessary [10].

Psychotherapeutic interventions—whether formal or brief, and by referral or the prescriber—can be helpful. It is often useful to separate medical management from psychotherapy to prevent medication discussions from dominating psychotherapy sessions [11]. However, psychotherapists and prescribers can both recommend and/or utilize anxiety management techniques, coping skills (to cope with stress without BZDs), relaxation techniques, breathing exercises, counseling, physical exercise, massage therapy, motivation enhancement, chemical dependence counseling, and CBT techniques. Cognitive behavioral interventions have been found to help facilitate the discontinuation of BZDs and reduce risk of relapse—especially in combination with a supervised taper [1,8,11,38,50,51]. Psychotherapies should remain supportive and cognitive-behavioral, focusing on “coping with protracted withdrawal symptoms, repairing relationships and learning to function without reliance on psychoactive drugs.” In early BZD abstinence or fragile patients, insight-oriented and psychodynamic psychotherapies risk mobilizing strong affects, memories, and emotions that may increase the risk of relapse [11]. CBT plus tapering has been studied the most and has the strongest evidence, and motivational enhancement also has some evidence [51]. CBT techniques focus on evaluating stressors and physical/emotional/behavioral consequences, and replace unhealthy/unrealistic thoughts (e.g., “BZDs are the only thing that will work,” “nothing else will help,” “if you take away my BZD, I’ll have a panic attack and die”) with healthy/realistic thoughts (e.g., “there are many healthy ways that can improve my sleep,” “even if the next treatment fails, my doctor will work with me until my anxiety is controlled,” “I know my anxiety will worsen somewhat at first and panic attacks are uncomfortable, but I can get through them and master my feelings”). “The discovery that a panic attack can be controlled without resorting to a tablet is a great boost to self-confidence” [5]. While most CBT techniques can be helpful during BZD withdrawal, exposure therapy is less effective during BZD use than after withdrawal, likely due to BZD-induced cognitive effects [5].

### 4.2. Withdrawal

When BZDs are suddenly discontinued in tolerant patients, they become exposed to hypoactive inhibition of GABA and hyperactive excitation of glutamate [1,18]. This combination causes withdrawal symptoms, which often leads to the perception that baseline anxiety is worse without BZDs. Most patients who take BZDs long-term will experience clinically significant withdrawal symptoms [15,52]. Even patients who use low doses short-term (even as few as 3 weeks) may experience mild withdrawal [15,18]. There is also evidence to suggest that sporadic, non-continuous BZD use may sensitize patients to future withdrawal [18].

There are three types of BZD discontinuation symptoms: recurrence, rebound, and withdrawal.

Recurrence symptoms are identical to the symptoms for which BZDs were originally prescribed. It is common for both patients and providers to misinterpret rebound and withdrawal symptoms to be recurrence symptoms, leading patients to mistakenly believe these symptoms make up their permanent baseline condition without BZDs. Other than a few symptoms (e.g., seizures, delirium, paresthesia, hypersensitivity to light or sound), there are few withdrawal symptoms that are distinguishable from common anxiety symptoms [11,18].

Rebound symptoms are “the mirror image” of therapeutic effects of BZDs (e.g., worse anxiety, insomnia, and restlessness) [53]. These symptoms occur shortly after discontinuation, including between doses (especially with short half-life BZDs) [50]. This generally causes patients to be acutely aware of their next dose. Such craving is common, and may itself exacerbate symptoms of anxiety [1,52,54,55,56].

Withdrawal symptoms are idiosyncratic to drug classes, and are not present before the particular drug was first used. For BZDs, severe symptoms usually occur after abrupt discontinuation (e.g., seizures, arrhythmia, death), but more mild symptoms may also occur with gradual tapering (e.g., anxiety, insomnia, tremors) [53,54]. The course of withdrawal is different depending on half-life, and may be slower with elderly or liver disease patients [11,15,18,54]. Withdrawal symptoms (particularly protracted withdrawal) may be due to individual constitutional and psychological factors in addition to GABA-A receptor changes. “Determining whether symptoms that emerge during [BZD] taper are actually a recurrence of anxiety or are related to a withdrawal syndrome is often difficult” [50].

While most patients taking long-term BZDs experience withdrawal symptoms upon discontinuation, short half-life BZDs can cause earlier and more severe withdrawal [50]. Table 2 summarizes the characteristics of various common BZDs [1,15,39,57]. Short half-life BZD withdrawal usually starts within 6–24 h, peaks in intensity at 1–4 days, and significantly improves by 4–5 days. Long half-life BZD withdrawal usually starts within 1–7 days, peaks in intensity at 5–14 days, and significantly improves by 3–4 weeks. In some patients, mild withdrawal symptoms may last for months to years (i.e., protracted withdrawal syndrome) [48,58]. With gradual reduction (over 2–4 months), the withdrawal of short-term and long-term BZDs are more similar and less severe [50].

Severe withdrawal symptoms are associated with higher doses, higher potency, shorter action and/or half-life, more rapid discontinuation, the elderly, organic brain damage (even minimal brain damage, which may be common in the elderly), SUDs, anxiety disorders, personality disorders, higher neuroticism, more baseline psychological distress, more baseline behavioral inhibition due to anxiety, less social support satisfaction, lower quality of life, and lower education level [1,11,18,47]. Unfortunately, some of these factors (e.g., comorbid substance use, anxiety and personality disorders) also correlate with increased BZD prescriptions (see above). Though rare, complicated BZD withdrawal (like with alcohol) may cause psychosis, tonic-clonic seizures (occurring in 20–30% of patients with untreated withdrawal), delirium, and/or death [11,15,53,54].

In a study [55] of 79 PTSD patients receiving long-term alprazolam (2–9 mg/day for 1–5 years) who attempted tapering or discontinuing the medication, 34 had withdrawal symptoms and 8 had severe withdrawal symptoms. Severe withdrawal symptoms included anxiety (8/8), sleep disturbances (8/8), nightmares or intrusive thoughts (8/8), rage reactions (6/8), homicidal ideations (6/8), suicidal ideations (4/8), and dissociative reactions (4/8). All 8 participants had symptoms that had never occurred before alprazolam treatment or were rebound symptoms of a severity never experienced before. Of note, all 8 patients with severe withdrawal had a prior history of alcohol and/or BZD use disorders. Because severe withdrawal reactions occurred even with gradual tapering, the authors suggested that the withdrawal syndrome with alprazolam may be more severe than with other BZDs. The study cautioned against starting BZDs without considering the risk of withdrawal, even with gradual taper.

There are three strategies for preventing life-threatening or distressing withdrawal when discontinuing BZDs: slowly decrease the dose (tapering), substitute a long-acting BZD then slowly decrease the dose, and substitute a long-acting barbiturate then slowly decrease the dose.

### 4.3. Tapering

A taper is necessary for safe and successful BZD discontinuation. Weaning from benzodiazepines should be done systematically with a full appreciation of the potentially-fatal consequences of abrupt cessation. Providers should reduce BZDs with patients’ consent and cooperation when possible [10], though unilateral decisions to taper BZDs may be appropriate when the risks of continued prescriptions outweigh the benefits. A tapering schedule plan should be made initially and then modified according to frequent evaluation. Those that reduce their BZD dose slowly often deny any withdrawal symptoms, and have improvements in mental health (including sleep and cognition) and physical health (including requiring fewer medical consultations) [1,49].

Hospitalization may be appropriate is cases with severe panic, suicidal ideation, or confusion [18], those with a history of complicated withdrawal (i.e., hallucinosis, seizures, delirium), or with compounding comorbidities (e.g., epilepsy, arrhythmias). While inpatient BZD withdrawal is typically easier and more rapid (usually days, or at most a few weeks), outpatient withdrawal is more likely to allow patients to build alternative coping skills gradually [5], which can improve future success.

Withdrawal symptoms can be lessened or avoided entirely with gradual tapering that considers dose, potency, duration of BZD use, duration of taper, rate of taper, frequency/timing of dosing, and psychosocial factors (e.g., lifestyle, personality, stressors, and support). For example, because withdrawal insomnia is a major concern, taking higher doses or the total BZD dose at night can increase discontinuation tolerance [1,5]. A long duration of taper is generally thought to best, with many recommending tapering over at least 2–4 months [24,50]. The whole process of BZD withdrawal may take months. Rapid withdrawal can increase distress and decrease success, but some argue that withdrawing at too slow of a rate may prolong distress. During withdrawal, symptoms commonly wax and wane, varying in severity and type. “Patients need not be discouraged by these wave-like recurrences; typically ‘windows’ of normality, when the patient feels well for hours or days, appear after some weeks, and over time these ‘windows’ enlarge while discomfort slowly regresses” [5].

Regarding rate of tapering, there are varying recommendations. Length of time of tapering is unpredictable, ranging from a few weeks to months depending on dose, duration, type of drug, patient’s physical health, and concomitant psychopathology. Usually the rate of discontinuation decreases over time as most withdrawal symptoms occur in the last half of tapering. Patients are usually able to tolerate gradual tapering without withdrawal symptoms until they are at 10–20% of their highest dose [11]. For the most effective and safest (especially in outpatient settings), most recommend tapering no faster than 25% of the total daily dose per week [1,49,59]. Because the end of the taper can often be most difficult, some recommend 25% daily dose reductions per week until (1) there is a 50% reduction in the original total daily dose, after which slow the rate even further [50]; (2) there is a 75% reduction in the original total daily dose, after which decrease the remaining dose one-eighth to one-tenth per week until discontinued; or (3) 4 weeks have passed, after which space out the further reductions for a total discontinuation period of 12 weeks [47]. While tapering one half tablet daily every 2 weeks can be effective for those who have taken BZDs for less than a year, more rapid tapering can be effective for those who have taken BZDs for over a year (e.g., 0.5 mg/week for alprazolam, 1 mg/week for lorazepam) [15]. Ideally, the patient should be in control of the reduction rate with informed consent and support from the provider [1,8]. A useful technique is to offer 2–3 reasonable options for rate/dose/frequency changes (but not an option for no change) and allow the patient to choose.

Original dose may influence tapering rates. For those with therapeutic dose dependence, a slow tapering rate of one-eighth to one-tenth of the daily dose every 1–2 weeks with optimal time for withdrawal from 6–8 weeks up to 1 year is helpful. For those taking less than 20 mg of diazepam equivalents daily, patients usually tolerate decreases of 1 mg every 1–2 weeks. For patients taking 20–40 mg, patients usually tolerate decreases of 2 mg every 1–2 weeks until the daily dosage is 4–5 mg, after which decreases of 0.5 mg every 1–2 weeks are better tolerated. Stopping the last 4–5 mg are often particularly difficult for patients (usually because of fears about how they will cope without the medication), but they are generally surprised by how easy it actually is. For patients taking larger doses (some abusers take over 0.5 g of diazepam equivalent dose daily), patients usually tolerate decreases of 10 mg every 2–3 weeks. For those with high-dose BZD dependence in the presence of polysubstance use, Ashton recommends inpatient detoxification for the primary drug and BZD conversion to diazepam with withdrawal over 2–3 weeks [1,5].

During tapering, withdrawal severity can be measured with the Benzodiazepine Withdrawal Symptom Questionnaire (BWSQ), which has demonstrated high test–retest reliability, construct validity, and predictive validity for monitoring BZD withdrawal. Low BWSQ scores during the end of tapering predict less or no use of BZDs during the year after discontinuation [11]. BWSQ assesses multiple signs/symptoms, including: dissociation; noise, light, smell, and touch sensitivity; peculiar taste in the mouth; muscle pain and twitching; paresthesia; dizziness; lightheadedness; feeling sick; dysphoria; sore eyes; motion sickness; hallucinations; feeling unable to control your movements; loss of memory; and loss of appetite [60].

### 4.4. Substitution or Adjuvant Pharmacotherapy for Withdrawal

To increase safety and decrease distress during discontinuation, many prescribers substitute one BZD for another longer-acting cross-tolerant sedative-hypnotic and/or add additional medications to control withdrawal symptoms. Tapering the current BZD may be useful for long-acting agents, for those without other substance use and for those who are likely to adhere to dosing regiments. However, in other cases, substitution of a long-acting BZD or a barbiturate can be particularly useful.

Because withdrawal is worse with short-acting BZDs, transition to long-acting BZDs (e.g., diazepam, clonazepam, chlordiazepoxide) can facilitate eventual discontinuation of all BZDs. Long-acting BZD substitution increases BZD discontinuation successfulness, especially for those with both alcohol and BZD dependence. Discontinuation of BZDs can be facilitated by changing to long half-life BZDs (e.g., diazepam) and slowly tapering the dose [10]. For some patients taking a high-potency BZD, Ashton recommends converting to diazepam due its slow elimination. This conversion can be done in stages by cross-tapering based on equivalent doses [1]. It is recommended to convert to diazepam and reduce the dose by about 10 mg every 2–3 days or as the patient tolerates and, when the daily dose is about 20 mg, the rate of taper can be decreased to about 5 mg every few days [61]. It then becomes more tolerable and safer for the patient to taper off the lower potency, longer acting diazepam. Diazepam is useful for withdrawal due to available formulations of scored tablets of 2, 5, and 10 mg [5]. Another advantage of diazepam is that it is more lipophilic than chlordiazepoxide, and therefore has a more rapid onset of action (which makes it favorable to prevent severe withdrawal) [61]. Conversion to diazepam is usually sufficient to prevent seizures, but some recommend carbamazepine [1]. One disadvantage of diazepam is that it is low potency. While both will prevent severe withdrawal, for high potency BZD substitution, clonazepam may be better tolerated than low-potency long-acting BZDs. Just as long-acting opioid methadone is used to transition patients off short-acting heroin, long-acting clonazepam can be used to transition patients off short-acting alprazolam.

Substitution of long-acting barbiturates can replace BZDs to facilitate discontinuation due to slower clearance [18]. Table 3 summarizes BZD to barbiturate dose conversions. Barbiturates have the advantages of little fluctuation of blood levels between doses and of being effective on GABA-A receptors that have become BZD-insensitive due to long-term BZD use. Low-dose BZD discontinuation can be facilitated by discontinuing the BZD, starting phenobarbital 200 mg/day on the first day, and slowly tapering as tolerated. High-dose BZD discontinuation can be facilitated with conversion to phenobarbital which can then be tapered 30 mg/day. Before receiving each dose of phenobarbital, the patient should be examined for signs of barbiturate toxicity: sustained nystagmus (the most reliable), slurred speech or unsteady gait. If nystagmus is observed, the scheduled dose should be withheld. If all three signs are observed, the next two doses should be withheld and the total daily dosage for the next day should be decreased by 50%. If there are signs of sedative-hypnotic withdrawal, the daily dose is increased 50%. If there are no signs of intoxication or withdrawal, the phenobarbital taper is again continued at a rate of 30 mg/day. The major disadvantage of barbiturates is a lower therapeutic window that may make clinicians nervous about prescribing and monitoring it as outpatients. However, phenobarbital intoxication is not known to produce euphoria or behavioral disinhibition, which may make patients more likely to view it as a medication [11]. Nevertheless, barbiturate substitution is typically reserved for inpatient BZD discontinuation.

Adjuvant medication may be helpful during BZD withdrawal, but no medications have consistently demonstrated relief of general withdrawal. No adjuvant medications have been proven effective in attenuating BZD withdrawal symptoms. Among those medications studied have been antidepressants, beta blockers, buspirone, anticonvulsants (e.g., gabapentin, carbamazepine), flumazenil, captodiamine, and progesterone. Of these, carbamazepine has the strongest—though still relatively weak [5]—evidence of promise for BZD withdrawal [62,63]. While non-BZD GABA receptor agonists (e.g., zolpidem, zaleplon, eszopiclone) have proven to relieve BZD withdrawal symptoms, they are contraindicated due to having the same disadvantages as BZD (i.e., dependence and abuse) [1]. Nevertheless, some occasional adjuvant medications may be helpful during discontinuation [1,5]. Several medications have been used for temporary symptom relief during BZD withdrawal: propranolol, non-BZD hypnotics, TCAs, clonidine, and analgesics. These drugs help control certain symptoms but do not alleviate overall withdrawal [48]. If autonomic hyperactivity is present (e.g., tachycardia), beta blockers (e.g., propranolol) or 2-adrenergic agonists (e.g., clonidine) may be useful adjuncts [11]. Antidepressants can help with dysphoria [48]. Low doses of TCAs may help for anxiety, insomnia, and depression. However, SSRIs may precipitate acute anxiety in some cases. Beta-blockers such as propranolol may help with palpitations, tremors, and muscle twitches but have little effect on overall states. Buspirone, clonidine, nifedipine, and alpidem have demonstrated no benefit, and may sometimes worsen withdrawal symptoms [5].

### 4.5. Replacement Pharmacotherapy for Anxiety and/or Insomnia

See Section 3.4 for evidence-based alternatives to sedative-hypnotics. Again, psychotherapy is the gold standard treatment for either anxiety or insomnia. As far as pharmacological agents, serotonergic agents and a variety of non-sedative-hypnotic agents have the strongest evidence for anxiety and insomnia, respectively. When prescribers and patients find the need for fast-acting medication, Table 1 presents several alternatives. However, treatments that enhance patient coping skills and build confidence should always be emphasized and preferable to exogenous means of sedation. While the lack of immediate response/remission can frustrate patients and providers, prescribers should not resort to prescribing ineffective or harmful medications just to satisfy their own feelings of helplessness.

## 5. Conclusions

A risk-benefit analysis of continued BZD use should be examined by providers before prescribing BZDs. In patients where there is evidence of dangerous side effects, misuse (e.g., drug screen positive for other substances, BZDs obtained from multiple providers, early refills, taking more than prescribed, suspicious claims of lost or stolen prescriptions), or diversion (e.g., drug screen negative for BZDs), providers should discontinue BZDs as rapidly as safely possible, whether the patient agrees or not. However, when there is lack of harm and patients refuse to decrease or discontinue BZDs despite education about evidence of inefficacy and harm, different prescribers have different philosophies and may implement different plans on a case-by-case basis. Some would argue that the medication should be discontinued, whether the patient agrees or not, to prevent harm (which is often insidious, hidden from both patients and providers) from occurring and because providers should not support treatments that they themselves believe to be ineffective (non-maleficence). However, others would argue for approaching the problem as they would approach patients who engage in other harmful behaviors (e.g., smoking, unhealthy eating), with building of a therapeutic alliance, continued psychoeducation, and supportive promotion of stages of change. Some argue that this is a different situation because with behaviors like smoking or unhealthy eating, providers are not prescribing the cigarettes or junk food. The counterargument is that if a provider does not work through the stages of change, the patient is simply apt to find another provider without qualms about prescribing BZDs—one who is likely not going to help the patient eventually decrease or discontinue BZDs. Debates like this can go on in perpetuity, but it is important that prescribers always discuss with the patient and document their reasoning. If stipulations are used (e.g., provider will not prescribe BZDs if the patient does not get random or regular drug screens, tests positive for other substances, does not engage in evidence-based treatments like psychotherapy and SSRIs), they should be discussed with the patient and documented.

## Figures and Tables

**Table 1:** Benzodiazepine risks, benefits, and alternatives.

Risks	Benefits	Alternatives
Physical adverse effects (e.g., commonly dizziness, slurred speech, and psychomotor impairment; most seriously overdose, withdrawal, falls, autonomic instability, seizures, hepatotoxicity, respiratory depression, and death) Cognitive adverse effects (e.g., commonly drowsiness, inattention; most seriously confusion, amnesia, hallucinations, delirium and coma) Emotional adverse effects (e.g., commonly depression, irritability, anxiety; most seriously lability) Behavioral adverse effects (e.g., commonly disinhibition, insomnia and avoidance; most seriously suicidality, violence and abuse) Other (e.g., teratogenicity, breast-feeding risks, drug-drug interactions)	Short-term anxiolytic effects Short-term hypnotic effects Fast-acting Can be prescribed as needed	**Acute/Fast-acting Treatment** Behavioral techniques Psychotherapy Sedating antidepressants (e.g., trazodone, mirtazapine, tricyclics) Adrenergic inhibitors (e.g., prazosin, propranolol) Antihistamines (e.g., hydroxyzine) Anticonvulsants (e.g., gabapentin, pregabalin) Antipsychotics (e.g., quetiapine, olanzapine, risperidone) **Chronic Treatment** Behavioral techniques Psychotherapy Serotonergic agents (e.g., antidepressants, buspirone) Antipsychotics Anticonvulsants (e.g., lamotrigine)

**Table 2:** Approximate benzodiazepine characteristics and equivalent doses (therapeutic equivalent doses are approximate due to clinical potency varying between individuals).

	Onset (hours)	Action Duration	Half-Life (hours)	Potency	Equivalent Doses (mg)
flurazepam	1	Long	* 40–250	low	15–30
chlordiazepoxide	1.5	Long	* 36–200	low	10–25
diazepam	1	Long	* 36–200	low	5–10
clorazepate	1	Long	* 36–200	low	7.5–15
clonazepam	1	Long	18–50	high	0.25–0.5
temazepam	0.5	Intermediate	8–22	low	30
lorazepam	2	Intermediate	10–20	high	1
oxazepam	3	Short	4–15	low	15–20
alprazolam	1	Short	6–12	high	0.5
triazolam	0.5	Short	2–5	high	0.25–0.5

* active metabolites.

**Table 3:** Phenobarbital withdrawal equivalents of benzodiazepines (withdrawal equivalent doses are not the same as therapeutic equivalent doses).

	Dose Equal to 30 mg of Phenobarbital (mg)	Phenobarbital Conversion Constant
flurazepam	15	2
chlordiazepoxide	25	1.2
diazepam	10	3
clorazepate	7.5	4
clonazepam	2	15
temazepam	15	2
lorazepam	2	15
oxazepam	10	3
alprazolam	1	30
triazolam	0.25	120
